# Platelet activation: a promoter for psoriasis and its comorbidity, cardiovascular disease

**DOI:** 10.3389/fimmu.2023.1238647

**Published:** 2023-08-15

**Authors:** Ziqi Jiang, Xiaoran Jiang, Aijun Chen, Wenyan He

**Affiliations:** ^1^ Department of Dermatology, The First Affiliated Hospital of Chongqing Medical University, Chongqing, China; ^2^ The First Clinical College, Chongqing Medical University, Chongqing, China

**Keywords:** platelet, psoriasis, cardiovascular disease, leukocyte, endothelial cell, cytokines

## Abstract

Psoriasis is a chronic inflammatory skin disease with a prevalence of 0.14% to 1.99%. The underlying pathology is mainly driven by the abnormal immune responses including activation of Th1, Th17, Th22 cells and secretion of cytokines. Patients with psoriasis are more likely to develop cardiovascular disease (CVD) which has been well recognized as a comorbidity of psoriasis. As mediators of hemostasis and thromboinflammation, platelets play an important part in CVD. However, less is known about their pathophysiological contribution to psoriasis and psoriasis-associated CVD. A comprehensive understanding of the role of platelet activation in psoriasis might pave the path for more accurate prediction of cardiovascular (CV) risk and provide new strategies for psoriasis management, which alleviates the increased CV burden associated with psoriasis. Here we review the available evidence about the biomarkers and mechanisms of platelet activation in psoriasis and the role of platelet activation in intriguing the common comorbidity, CVD. We further discussed the implications and efficacy of antiplatelet therapies in the treatment of psoriasis and prevention of psoriasis-associated CVD.

## 1 Introduction

Psoriasis is a chronic immune-mediated inflammatory skin disease, with a prevalence in different populations varying from 0.14% to 1.99% (1). The classical lesion of psoriasis is a well-demarcated raised, red plaque with a white scaly surface, occurring on the elbows, knees, scalp, and even any skin surface of the body. According to WHO report, psoriasis is associated with substantial psychological and economic burden (2). Despite this, the molecular mechanisms underlying the pathogenesis of psoriasis are not fully understood. External stimuli and genetic factors co-contribute to the activation of dendritic cells (DCs) and thus initiate the inflammatory cascade. The increased expression of cytokines by DCs including interleukin (IL)-12 and IL-23 induce T cell activation and differentiation into T helper (Th) cells. A subsequent inflammation and keratinocyte proliferation are aggravated by secreted cytokines from Th1, Th17 and Th22 cells, which ultimately lead to psoriasis progression (3–6).

It is well established that psoriasis is associated with a number of comorbidities including psoriatic arthritis, psychological illnesses, inflammatory bowel diseases, and cardiovascular diseases (CVD) (7, 8). Data have shown that psoriasis patients, especially those with >10% body surface area affected or a long duration or candidates for systemic therapy or phototherapy, are at increased risk for CVD including stroke, myocardial infarction (MI), and cardiovascular (CV) death (9–12). In this context, the guideline issued by the American College of Cardiology (ACC)/American Heart Association (AHA) advocates psoriasis diagnosis into CV risk prediction, without specifying a psoriasis severity threshold (13).

Platelets are anucleate blood cells generated from megakaryocytes in the bone marrow. With mRNAs and platelet granules such as α-granules and dense granules inherited from megakaryocytes, and various glycoproteins (GPs) on cell membrane, platelets exert multiple functions in hemorrhagic, thrombotic, and inflammatory diseases (14, 15). An increasing body of evidence suggests that platelet hyperactivation is induced by the abnormal immune system in psoriasis patients, and excessive activation of platelets in return aggravates the inflammation, which results in a vicious cycle in the etiopathogenesis of psoriasis (16). On the other hand, platelet dysfunction is involved in the pathogenesis of CVD including atherosclerosis, coronary thrombosis, stroke, and MI (17, 18). A Gene Ontology (GO) enrichment analysis showed that platelet aggregation and platelet activation terms were in the top five biological process terms in the psoriasis group with CVD risk factors (19), highlighting the important role of platelets in psoriasis-associated CVD.

This review mainly focuses on the biomarkers, mechanisms, and effects of platelet activation in psoriasis and CVD. We discuss how activated platelets contribute to psoriasis progression and initiate psoriasis-associated CVD, thereby providing an outlook for antiplatelet therapies in psoriasis in the future.

## 2 Biomarkers of platelet activation associated with psoriasis and CVD

### 2.1 Mean platelet volume & platelet distribution width

Platelet volume has been proven to be associated with various diseases such as CVD, systemic lupus erythematosus, and Alzheimer’s disease. Recognized as a marker of proliferation, metabolism, and thrombopoiesis of megakaryocytes in bone marrow and platelet functions, platelet volume is typically reflected by mean platelet volume (MPV) and platelet distribution width (PDW).

MPV, representing platelet morphological changes, is a marker indicating the activation and functions of platelets. Several studies have found patients with psoriasis are usually accompanied by a higher level of MPV when compared with healthy controls (**Table 1**) (20–30). Most of these studies included all types of psoriasis patients (20–24, 28), while some focused on patients with psoriasis vulgaris or psoriatic arthritis and revealed a similar elevated MPV level in these patients (25–27, 29, 30). Though several studies showed negative or no correlation between MPV and the psoriasis area and severity index (PASI) scores (27–30), more studies demonstrated that MPV levels were positively correlated with PASI scores, suggesting that MPV is an indicator of disease severity (20–26). Additionally, MPV levels after biological therapies were drastically decreased, aligning well with the PASI scores (30). Interestingly, MPV levels are higher in patients who developed CVD than non-CVD controls, and the risk of death or MI is higher in those CVD patients with larger MPV, which makes MPV an important predictive factor of diagnosis and prognosis in CVD (48). Therefore, using MPV as a predictive marker for developing CVD in psoriatic patients would be of value to investigate.

**Table 1:** Biomarkers of platelet activation in psoriasis.

Biomarker		Levels in psoriasis	References
Platelet volume	MPV	Higher levels vs. healthy controls, correlated positively with PASI	(20–26)
		Higher levels vs. healthy controls, not correlated with PASI	(27, 28)
		Higher levels vs. healthy controls, correlated negatively with PASI	(29, 30)
		No significant difference vs. healthy controls	(31, 32)
		Decreased levels after biological therapy	(30)
	PDW	Higher levels vs. healthy controls, not correlated with PASI	(24, 25, 27)
		No significant difference vs. healthy controls	(31)
P-selectin		Higher levels vs. healthy controls, correlated positively with PASI	(24, 33–35)
		Higher levels vs. healthy controls, not correlated with PASI	(36–38)
		Higher levels vs. healthy controls	(39)
		No significant difference vs. healthy controls	(40)
		Decreased after effective therapy	(34, 35, 37)
PDMPs		Higher levels vs. healthy controls, correlated positively with PASI	(24, 37, 41)
		Higher levels vs. healthy controls	(42, 43)
		Decreased after topical or anti-TNF-α therapy	(37, 44)
		No significant difference after anti-IL-12/23 therapy	(42)
PF4		Higher levels vs. healthy controls, correlated positively with PASI, decreased after topical steroids therapy	(32)
IFN-induced transcripts		Higher levels of IFIT1, IFIT3, IFIT2, IFIT5, IFITM3, OAS2, STAT1 vs. healthy controls, correlated positively with PASI	(45)
TSP-1		Decreased levels vs. healthy controls, correlated negatively with disease activity	(46, 47)

MPV, mean platelet volume; PDW, platelet distribution width; PDMP, platelet-derived microparticle; PF4, platelet factor 4; IFN, interferon; TSP-1, thrombospondin-1; PASI, psoriasis area and severity index; vs., versus.

PDW is another important biomarker representing heterogeneity of platelet volume and platelet activation. A higher PDW level means more platelets in different sizes in the peripheral blood. Usually, PDW is positively related to MPV. Studies revealed higher PDW levels in patients with psoriasis, compared to the healthy controls (**Table 1**) (24, 25, 27). Nevertheless, PDW showed no significant correlation with PASI scores in these studies, indicating that it was not associated with disease severity. Similar to MPV, PDW levels are also increased in atherosclerosis, coronary artery disease, and cerebrovascular disease, and can predict the occurrence of cardiac death and infarction recurrence in patients with acute MI (49, 50). Based on these findings, elevated levels of MPV and PDW, which represent platelet hyperactivation, are critical indicators in the development of CVD and conducive to predictive of CV risk in psoriasis patients.

### 2.2 P-selectin

P-selectin, also referred to as CD62P or granular membrane protein-140, is a GP found in the α-granules of platelets and the Weibel-Palade bodies of endothelial cells. The P-selectin in plasma, called soluble P-selectin (sP-selectin), is considered to originate from platelets only. Activated platelets trigger membrane surface expression of P-selectin and its secretion into the plasma (24), which makes P-selectin a marker of platelet activation. P-selectin has been shown to mediate the interaction between activated platelets, endothelial cells and neutrophils, monocytes, eosinophils, T lymphocyte subsets (51). This essential role contributes to the inflammatory infiltration in psoriasis patients. Most studies, except one study conducted by Kwiek et al. in 2017 (40), found that levels of P-selectin, especially sP-selectin, were higher in psoriasis patients than healthy controls (**Table 1**) (33–39). Besides, sP-selectin levels were reduced significantly after treatments including clobetasol propionate, calcipotriol, narrow-band ultraviolet B (NB-UVB) and biological drugs (34, 35, 37). Intriguingly, four of these studies showed a positive correlation between sP-selectin and PASI scores (24, 33–35), while the other three studies showed no correlation (36–38), suggesting a controversial relationship between sP-selectin and PASI scores, which may be related to the demographic characteristics or disease types (psoriasis vulgaris, psoriatic arthritis, pustular psoriasis, etc.) of patients and requires further studies. The raised sP-selectin in a wide variety of acute and chronic CVD has been confirmed previously (52). So P-selectin levels can show a certain relationship between CVD, psoriasis and platelet activation, and can serve as a special marker of irreversible platelet activation and an indicator for treatment efficacy in psoriasis and psoriasis-associated CVD.

### 2.3 Platelet-derived microparticles

It has been found that activated platelets secrete platelet-derived microparticles (PDMPs) in an exocytotic budding way, thus PDMP is supposed to be a biomarker of platelet activation. PDMPs are membrane vesicles that can carry various molecules such as cytokines and lipid mediators. Aside from the classical procoagulant property, PDMPs also have regulatory effects on immune function and intracellular communication. Benefited from the special structural characteristics, PDMPs can cross over from blood into the synovial fluid, lymph, and bone marrow (53). Levels of PDMPs were elevated in psoriasis patients (**Table 1**) (24, 37, 41–43), and a positive relationship between PDMP levels and PASI scores, as well as MPV, PDW, and P-selectin, were observed (24). These changes in PDMP levels demonstrate that psoriasis severity is closely related to platelet activation. Moreover, PDMP levels were reduced after treatment with anti-tumor necrosis factor-α (TNF-α) agents or topical therapies, such as 0.05% clobetasol propionate and 0.005% calcipotriol (37, 44). Nevertheless, not all biological therapies (e.g.: IL-12/13 blockages) exerted inhibitory effect on PDMP levels (42). These data showed different efficacy in decreasing PDMP levels of various treatments, which may attribute to the numerous subtypes of PDMPs in different sizes and carrying different components. On the other hand, elevated PDMP concentrations are also observed in various CV conditions such as ischemic stroke, coronary artery disease, and hypertension, attributed to its role in adhesion, inflammation, procoagulation, and lipid deposition. Together, monitoring PDMP levels may have beneficial clinical implications in screening and prevention of psoriasis and CVD (54).

### 2.4 Platelet factor 4

Platelet factor 4 (PF4), also known as CXCL4, is a useful marker of platelet activation released from α-granules of activated platelets (55). PF4 is not only associated with platelet function, but also promotes recruitment and activation of different inflammatory cell types, and modulates the immune response. Significant higher PF4 levels were found in psoriasis patients when compared with healthy controls. Moreover, PF4 levels were correlated with PASI scores, and reduced after conventional therapies such as topical steroids (**Table 1**) (32). Additionally, PF4 was involved in the development and progression of atherosclerosis and other cardiovascular diseases (56, 57). Immunofluorescence studies by Gleissner et al. also revealed an increased expression of PF4 in human atherosclerotic plaques (58). In conclusion, PF4 is a proper marker of platelet activation associated with psoriasis and CVD, and elevated levels of PF4 suggests the essential role of platelet activation in these diseases, which still needs to be studied in depth.

### 2.5 Platelet IFN-induced transcripts

Interferon (IFN) is one of the central factors in the pathogenesis of psoriasis inflammation. IFN-α and IFN-β initiate myeloid DCs (mDCs) activation and inflammatory cascade in psoriasis, while IFN-γ has been considered to play a role in determining disease severity and therapy evaluation (59). To investigate the association between IFN and activated platelet phenotype, Garshick et al. performed platelet RNA sequencing in psoriasis patients and healthy controls, and found IFN signaling was the top upregulated pathway in psoriasis platelets. Moreover, they demonstrated that expression levels of platelet IFN-induced transcripts (e.g.: IFIT1, IFIT3, IFIT2, IFIT5, IFITM3, OAS2, STAT1, etc.) correlated with PASI scores, circulating IFN-γ and IL-17A levels, and could be increased following stimulation with the combination of IFN-γ and IL-17A (**Table 1**) (45). Furthermore, study by Zou et al. suggested that some of these IFN-induced transcripts (STAT1, IFIT1, and IFIT3) were key genes involved in psoriasis development (60). Higher levels of STAT1 were confirmed in psoriatic skin and STAT1 phosphorylation was increased following IFN-α or IFN-γ stimulation (61–65). In addition, Huang’s study revealed elevated levels of OAS1, OAS2, and OAS3 in psoriatic skin and serum and these IFN-induced enzymes decreased after biological therapies (66). Although it is not known to what extent these elevated IFN-related transcripts levels in psoriatic skin and serum could be attributed to platelets, studies collectively suggest that the increased IFN-induced transcripts levels of platelet can represent platelet activation and probably make platelet an important factor in IFN-induced inflammation in psoriasis. Apart from the role in psoriasis pathogenesis, IFN-β is proved to inhibit angiogenesis and arteriogenesis, while IFN-γ can promote development of MI and stroke, the two main atherosclerotic diseases (67, 68). Both of them are highly expressed in atherosclerotic lesions, but whether platelet IFN-induced transcripts play an equally important role in CVD as in psoriasis remains to be explored.

### 2.6 Thrombospondin-1

Thrombospondin-1 (TSP-1) is a homotrimeric, multidomain glycoprotein that is released from α-granules of activated platelets, present in the extracellular matrix, and circulates in plasma. TSP-1 induces platelet activation and aggregation through several pathways including CD36-dependent inhibition of the cAMP/protein kinase A signaling cascade and/or blocking the antithrombotic activity of nitric oxide/cGMP signaling (69, 70). In accordance with its functions, overexpression of TSP-1 was found in a series of CVD such as MI, cardiac hypertrophy, heart failure, angiogenesis, and atherosclerosis (71). Intriguingly, keratinocytes from psoriatic skin exhibited a reduced level of TSP-1 (46), and TSP-1 expression in psoriasis patients was inversely correlated with disease activity, which was related to the dysregulated expression of TSP-1 in the immune cells and inhibition of Th17 differentiation (**Table 1**) (47). However, the role of TSP-1 as a biomarker of platelet activation in psoriasis and CVD and as a link between the two diseases is still inconclusive, and is deserved to explore.

## 3 Multiple factors intrigue the activation of platelets in psoriasis

### 3.1 Endothelial damage

Although not completely implicated in the pathogenesis of psoriasis, the impaired endothelial function in psoriasis progression has been proven. Previous studies investigated the endothelial dysfunction via measuring circulating biomarkers (asymmetrical dimethylarginine, oxidized LDL, and endothelial progenitor cells) and vascular markers (pulse wave velocity, flow-mediated dilation, nitroglycerine-induced vasodilation, and aortic stiffness parameters), and results appeared in line with the hypothesis that endothelial functions in psoriasis patients were significantly damaged (72, 73). It is well known that damaged endothelium can initiate the platelet activation cascade. In brief, at the sites of endothelial damage, platelets are activated and adhere to the subendothelial extracellular matrix, the main components of which are fibrillar collagens type I and III. With the help of GPIbα on platelet membrane, von Willebrand factor (vWF) and fibrillar collagens, fibrinogen bridging mediates aggregation of adjacent platelets (45, 74). Consequently, available data above suggest that endothelial damage in psoriasis initiate platelet activation and aggregation at the injured endothelium.

### 3.2 Cytokines stimuli

Psoriasis originates as a result of dysregulated interactions of innate and adaptive components of the immune system with resident cutaneous cell types. Plasmacytoid DCs (pDCs), keratinocytes, natural killer T cells, and macrophages produce cytokines such as TNF-α, IFN-α and IFN-β to activate mDCs. Subsequently, more cytokines (especially IL-12 and IL-23) are released to induce T cell activation and differentiation, followed by IL-17, IFN-γ, IL-6 and TNF-α secretion from Th cells (3, 4, 6). Interestingly, cytokines in this self-amplifying loop result in platelet activation. For example, Maione et al. found that platelets pre-treated with IL-17 exhibited an increased ADP-induced P-selectin expression and fibrinogen binding, representing excessive platelet activation and aggregation (75). Hot A et al. also revealed that supernatants of IL-17A-stimulated primary endothelial cells facilitated the aggregatory response in platelets (76). Similarly, IL-1β, IL-6 and IL-8 can exert a regulatory effect on platelet function (77–79). In addition, IL-9 is also increased in psoriasis patients, which not only promotes Th17-associated inflammation and angiogenesis in psoriasis, but also facilitates P-selectin expression and platelet activation via JAK2/STAT3 pathway in deep venous thrombosis (80, 81). Collectively, these observations indicate a strong positive effect of cytokines on platelet activation.

### 3.3 Platelet-activating factor secretion

Platelet-activating factor (PAF) is a potent phospholipid-derived mediator with multiple biological effects on platelet aggregation, vasodilation, bronchoconstriction, host defense, inflammatory responses, and skin barrier function (82–84). PAF is produced by numerous cell types, such as platelets, keratinocytes, endothelial cells, and monocytes, the role of which has been implicated in several diseases including ischemic stroke, MI, and inflammatory diseases (85). Increased PAF levels were detected in plasma and scales of psoriasis patients (86, 87). In addition, the key factors (arachidonic acid [AA] and eicosanoids) and enzymes (phospholipase A2 [PLA2]) for PAF formation were also increased in psoriatic tissue (88). PAF regulates Th17 cells and IL-17 axis functions and contributes to chemotaxis, aggregation, and degranulation of peripheral polymorphonuclear leukocytes, thus enhancing inflammation in psoriasis (particularly, pustular psoriasis) (87, 89, 90). In accordance with these results, PAF receptor antagonist PCA-4248 was proved to lower leukocyte accumulation in the skin and block psoriasis progression (90). As a strong activator of platelets, PAF has been shown to induce shape change and release of granules contents in human platelets (91). Moreover, PAF mediated platelet activation and aggregation through activation of protein kinase C (PKC) pathway, mobilization of intracellular Ca^2+^, stimulation of the phosphatidylinositol cycle, and increased release of AA (92, 93). The higher levels of PAF in psoriatic skin, along with its regulatory effect on platelets in other diseases, suggest that PAF is also an important mediator of platelet activation in psoriasis.

## 4 The common effects of activated platelets on psoriasis and CVD

### 4.1 Platelets facilitate leukocyte infiltration

Leukocyte migration is an essential step for both psoriasis and CVD, as it’s followed by recruitment of immune cells and inflammatory infiltration into lesional skin and blood vessels. Thus, microabscess formation and thromboinflammatory injury are representative manifestations of psoriasis and CVD, respectively.

Activated platelets are found to adhere to endothelial junctions and capture leukocytes via CD40-CD40 ligand (CD40L) cross-talk. CD40, belonging to the TNF receptor superfamily, is a co-stimulatory receptor which can recognize CD40L molecule. Typically, CD40L is translocated from intracellular region to platelet surface after platelet activation, followed by the interaction with CD40-expressing cells including immune cells and endothelial cells (94, 95). Denfeld et al. demonstrated that keratinocytes and endothelial cells in early and chronic psoriatic lesions exhibited higher CD40 levels. And CD40-CD40L cross-talk between platelets and keratinocytes could promote T cell and monocyte infiltration into the psoriatic plaques, via increasing intercellular cell adhesion molecule (ICAM)-1, Bcl-x, IL-8, CCL20, RANTES and MCP-1 levels (96). Similarly, circulating platelet-leukocyte aggregation, which leads to the development of both atherosclerosis initiation and plaque rupture, is also promoted by CD40L (particularly human platelet-bound or recombinant soluble CD40L) via eliciting the formation and tethering of net-like ultra-large vWF multimers on endothelium (97–99). In advanced atherosclerosis, foam cell formation and production of matrix-degrading metalloproteinase (MMPs) by activated macrophages are significantly induced by CD40-CD40L cross-talk and stromal cell derived factor 1 (SDF-1) following platelet activation, which will lead to plaque destabilization and rupture (100, 101).

P-selectin derived from activated platelets also contributes to leukocyte migration, which exhibits a synergistic effect with CD40-CD40L cross-talk. P-selectin glycoprotein ligand-1 (PSGL-1) is the major ligand for P-selectin and can be detected in almost all kinds of leukocytes. P-selectin-PSGL-1 binding is one of the factors which mediate the interaction between platelets and most leukocytes, including polymorphonuclear neutrophils (PMNs), monocytes/macrophages, and lymphocytes in psoriasis (102, 103). An earlier study by Ludwig et al. confirmed that activated platelets supported leukocyte rolling in skin microvasculature by forming leukocyte-platelet aggregates through P-selectin-PSGL-1 binding (104). A later study by Zuchtriegel et al. further explored the role of P-selectin-PSGL-1 cross-talk, and showed that platelets could work as “pathfinders” guiding these leukocytes to the sites of inflammation, which emphasized the critical role of P-selectin in platelets-guided leukocytes extravasation. Specifically, P-selectin-PSGL-1 binding regulated ERK1/2 MAPK pathway and induced the high-affinity conformation of surface-expressed β2 integrin in neutrophils (95). Upregulated β2 integrin, along with other adhesion molecules such as ICAM-1 and vascular cell adhesion molecule (VCAM)-1, further guides the extravasation of immune cells to the perivascular tissue (95). In CVD such as atherosclerosis, P-selectin-PSGL-1 cross-talk also mediates mechanical interaction between platelets and neutrophils (105). In this way, recruited neutrophils scans for activated platelets and fosters their migration to inflamed or injured vessel wall, resulting in thromboinflammatory injury (106).

**Figure 1:**
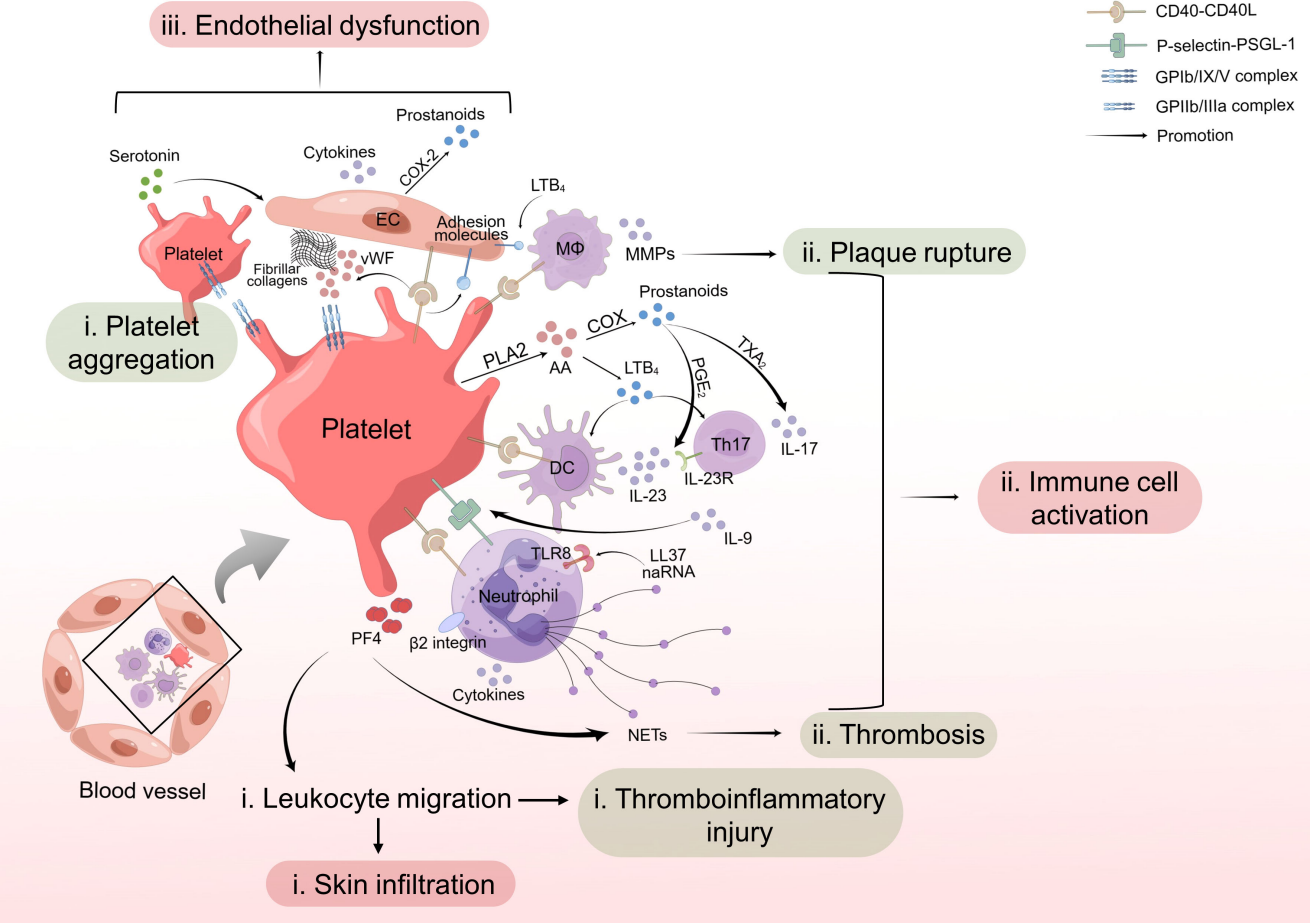
Role of platelet activation in psoriasis and cardiovascular disease. (i) Activated platelets interact with leukocytes through CD40-CD40L or P-selectin-PSGL-1 cross-talk and facilitate leukocyte migration, leading to skin infiltration and thromboinflammatory injury. (ii) Platelets contribute to activation of immune cells and increased release of NETs from PMNs, cytokines from DCs, Th17 cells and neutrophils, and MMPs from macrophages, further promoting thrombosis and plaque rupture. (iii) Platelet activation induces secretion of cytokines and adhesion molecules by endothelial cells, resulting in endothelial dysfunction. In summary, activated platelets work as promoters for psoriasis and psoriasis-associated CVD. CD40L, CD40 ligand; PSGL-1, P-selectin glycoprotein ligand-1; NET, neutrophil extracellular trap; PMN, polymorphonuclear neutrophil; DC, dendritic cell; EC, endothelial cell; MΦ, macrophage.

Studies by Herster et al. have found that platelet depletion could prevent PMNs infiltration into psoriatic skin in the imiquimod-induced mouse model (107), confirming the hypothesis that platelets are involved in leukocyte infiltration in the pathogenesis of psoriasis. Also, the recruitment of neutrophils to thrombi have been well demonstrated in previous studies (108). In summary, platelet-leukocyte aggregation and leukocyte infiltration induced by activated platelets in both psoriasis and CVD indicate platelet activation might be the link between the two diseases (**Figure 1i**).

### 4.2 Platelets contribute to immune cell activation

Researches on psoriasis and CVD have emphasized the role of abnormal immune cell activity in pathogenesis of the two diseases. Subsequent release of cytokines such as IL-17 following immune cell activation is regarded as a potential mechanistic link between psoriasis and CVD. The contribution of platelets to it has gradually been valued in the last decades.

Platelets are found by Gerdes et al. to enhance the differentiation of CD4+ T cells and the release of cytokines by Th1, Th17, and Treg subpopulations via both multiple chemokines and direct cell-cell contact (109). Later, Sanz-Martínez et al. further demonstrated that platelet activation in psoriasis stimulated the appearance of platelet-lymphocyte complexes and modulated the production of IL-17 (110). According to previous studies, AA and its metabolites, which can be produced from hyperactivated platelets with the activation of PLA2 and cyclooxygenase (COX), may contribute to platelet-induced immune cell activation (111–113). For example, 1) prostaglandin E_2_ (PGE_2_) facilitates the expansion of Th17 cells via up-regulated expression of IL-23 in DCs and IL-23 receptor subunit gene on Th17 cells (114, 115); 2) thromboxane A_2_ (TXA_2_) induces IL-17 secretion in psoriatic lesions (116); 3) leukotriene (LT)B_4_ promotes the recruitment of DCs and T cells and the production of cytokines including IL-23, IL-12 and IL-17, leading to intraepidermal microabscesses and hyperproliferation of epidermal cells (117, 118). However, there were no reports and literature of the relevant studies on AA metabolites directly derived from activated platelets in psoriasis. Although Vila et al. found an increased COX activity in platelets from psoriasis patients (119), suggesting a possible higher levels of AA metabolites released from psoriatic platelets, further studies are required to ascertain the effect of AA metabolites from these platelets, which has not yet been confirmed, in patients with psoriasis.

Neutrophil extracellular trap (NET) is another factor which plays an essential role in immune cell activation promoted by platelets. Hyperactivated PMNs secreting NETs and more cytokines, which promote keratinocytes proliferation and skin inflammation, have been observed in psoriasis patients in previous studies (120, 121). PAF was proved to mediate the promoting effect of activated platelets on NET formation in neutrophils in psoriasis patients (122). NET formation can also occur upon interaction of platelets with neutrophils via PF4 release in vasculitis (123, 124). Similarly in CVD, activated neutrophils produce NETs and consequently promote thrombosis via interactions with platelets (125). Low-density neutrophils co-localized with platelets induce increased NET formation and are strongly correlated with non-calcified coronary burden (122). In turn, NET-associated RNA (naRNA), along with the antimicrobial peptide LL37 that induces platelet aggregation and promotes thrombus formation (126, 127), triggers cytokine and NET release by PMNs through Toll-like receptor 8 (TLR8) signaling and aggravates inflammation in psoriasis and CVD (128).

In conclusion, platelet-intrigued dysfunction of T cells, DCs, neutrophils, macrophages or even more immune cells influences plaque instability of atherosclerosis, occurrence, and development of other CVD, and immune homeostasis of psoriasis, indicating that platelets work as a link between psoriasis and psoriasis-associated CVD through activating immune cells in different ways (**Figure 1ii**).

### 4.3 Platelets mediate endothelial dysfunction

Endothelial dysfunction is one of the fundamental factors for both psoriasis and CVD. As shown above, impaired vascular health has been observed in psoriasis patients (72, 73) and platelets might be involved in the process of endothelial damage in psoriasis. While platelet-triggered endothelial damage, especially endothelial inflammation, also results in abnormal fluid homeostasis and the development of atherosclerosis and other CVD (129). Therefore, platelet may be the perfect link between inflammatory skin diseases and systemic vascular change (130, 131).

An *In-vitro* study revealed that platelets isolated from psoriasis patients, but not from healthy controls, preferentially adhered to human aortic endothelial cells (HAECs) and induced proinflammatory transcriptional changes including upregulation of IL-8, IL-1β and COX-2, mainly through the tubular connections between platelets and HAECs (45). Kotowicz et al. found expressions of adhesion molecules in endothelial cells, including ICAM-1, VCAM-1 and E-selectin, were significantly enhanced by stimulation with CD40L (132), which was usually located in the membrane of activated platelets and bound with CD40 on endothelial cells to function. On the other hand, Kim et al. confirmed that integrin was the one of the most important proteins in the psoriasis group with high CVD risk through GO and IPA analysis (19). Consistent with this, the β1 and β2 integrin-dependent affinity of monocyte/macrophage to ICAM-1 and VCAM-1 on endothelial cells is enhanced by platelet-induced LTB_4_ and accelerate the adhesion of monocyte/macrophage to endothelium in atherosclerotic plaque (133). Similar to monocytes/macrophages, neutrophils also accumulate at the dysfunctional endothelium after plaque rupture via integrins, which will amplify platelet aggregation and blood coagulation (127, 134).

Besides, as an important mediator derived from platelets, serotonin may initiate the formation of gaps between endothelial cells and aggravate vascular leakage in several systems, such as the cerebral ventricular system and the synovial vasculature in arthritis (135). Studies by Cloutier et al. demonstrated that platelet serotonin accumulated via the serotonin transporter in the inflamed joints increased vascular permeability and further mediating inflammatory infiltration in an inflammatory arthritis mouse model (135). Intriguingly, serotonin reuptake inhibitors were found to improve the physical symptoms of psoriasis (136), suggesting there might be similar effects of platelet serotonin on endothelial function in psoriasis.

Collectively, platelets facilitated endothelial inflammation, amplified vascular permeability and induced adhesion molecule expression, contributing to endothelial dysfunction, finally aggravating psoriasis and inducing a higher CV risk in psoriasis patients (**Figure 1iii**).

## 5 Antiplatelet therapies serve as potential methods to prevent psoriasis-associated CVD

### 5.1 Low-dose aspirin therapy

Aspirin, also known as acetylsalicylic acid, has been universally recognized as a classical antiplatelet medication, because of its inhibitory effect on COX-1. It is a very common therapy applied to the prevention and treatment of CVD, as it could attenuate platelet-induced NET formation, reduce platelet-leukocyte aggregation and subsequent immune cell activation (137, 138), which may also help to abrogate inflammatory infiltration in psoriasis and atherosclerosis. As for its role in psoriasis, previous studies suggested that aspirin was able to reduce risk of MI and stroke in patients with higher plasma C-reactive protein levels (139), and to ameliorate vascular inflammation in psoriasis (140). Moreover, low-dose aspirin has also been shown to reduce serum TXB_2_ (the stable metabolite of TXA_2_) and improve endothelial inflammation induced by hyperactive platelets in psoriasis, via inhibition of COX-1 (45). Although the efficacy of aspirin on improvement of psoriatic skin manifestations remains unclear, antiplatelet and anti-inflammatory properties of aspirin make it a potential therapeutic approach for psoriasis and psoriasis-associated CVD. Nevertheless, drug risks should be carefully evaluated when using aspirin in combination with methotrexate and cyclosporine because aspirin may increase the toxicity of the other two drugs (141).

### 5.2 Statin therapy

It is well established that statins can drastically reduce CV risk because of their strong lipid-lowering effect. In addition, statins have been proven to attenuate platelet activation and aggregation in CVD (142). Various studies explored statin-specific molecular targets about platelets and their downstream effectors, including those involved in psoriasis pathogenesis which we have mentioned above. Firstly, statins (including simvastatin, atorvastatin and rosuvastatin, but not pravastatin) have been found to influence AA metabolism, via reducing platelet PLA2 phosphorylation and COX-1 activation, and to inhibit PG production as well as AA-induced platelet aggregation (143–146). Secondly, studies demonstrated that atorvastatin suppressed platelet CD40L expression and platelet activation with consequent reduction of leukocyte aggregation and MMP secretion (147, 148). Thirdly, expression of P-selectin and its major ligand, PSGL-1, were reduced by statin therapy (149), thus inhibiting the adhesion of activated platelets to leukocytes and extravasation of immune cells mediated by P-selectin-PSGL-1 interaction. However, it should be noted that different statins may have different effects on platelet activation according to their different molecular composition and variable impacts on signaling pathways, which calls for more research later on.

Considering the efficacy of statins in CVD treatment and the accumulation of evidence of accelerated CVD in psoriasis, earlier use of statins has been recommended in patients with psoriasis, particularly those with a ≥5% 10-year risk of atherosclerotic disease, by both the ACC/AHA guidelines and the American Academy of Dermatology/National Psoriasis Foundation (AAD/NPF) guidelines recently (9, 150). On the other hand, studies demonstrated that statins could significantly reduce PASI scores and improve disease severity in psoriasis (151, 152), indicating the nonnegligible role of statin therapy in both psoriasis and its comorbidity, CVD.

In addition, statins have been shown to increase the sensitivity of platelet cell membrane to aspirin treatment, which could consequently amplify the effect of aspirin on platelets (141). Psoriasis patients with higher CV risk usually need polydrug therapies, so the synergistic effect of aspirin and statins should be taken into account in disease management.

### 5.3 Classic psoriasis therapies

Apart from the conventional antiplatelet drugs, some therapies may have benefits beyond psoriasis in inhibiting platelet activation. Studies by Sanz-Martínez et al. demonstrated that psoriasis patients receiving anti-TNF-α therapy, one of the most common-used biological agents in psoriasis, showed a significantly lower risk of major adverse CV events compared to those receiving topical or oral and/or phototherapy agents (110). Decreased levels of platelet-lymphocyte complexes and PDMPs were found in responder group of patients (37, 44, 110), highlighting that anti-TNF-α therapy could reduce platelet activation and may mitigate CV risk in psoriasis.

However, the effects of other treatments for psoriasis on platelet activation are still inconclusive. For example, 1) secukinumab showed no effect on platelet/lymphocyte ratio (153); 2) anti-IL-12/23 treatment had no effect on PDMPs (42); 3) high concentrations of psoralen and ultraviolet A radiation (PUVA) promoted platelet aggregation *in vitro*, but caused no detectable abnormality in platelet function *in vivo* (154); 4) PUVA induced immune suppression in skin inflammation and apoptosis via activation of the PAF pathway (155), which led us to propose that PAF may also be involved in the effect of PUVA on platelet activation. Wu et al. found that patients with psoriasis who were treated with PUVA exhibited a higher CV risk than those treated with anti-TNF therapies (156), but no studies have been reported about effects of PUVA on CV risk in psoriasis patients compared with those who received no treatment. Prospective studies are still necessary to verify the effects of biological agents on more biomarkers of platelet activation such as MPV, PDW, and P-selectin in patients with psoriasis, and to further explore the role of PUVA in platelet activation.

### 5.4 Natural products

Recent studies showed that natural products extracted from herbal medicines may have synergistic effects to alleviate psoriasis and platelet activation. For example, curcumin, frequently employed as an anti-inflammatory agent in conventional medicine, could be a complementary therapy of psoriasis which reduced serum levels of IL-22 and PASI scores (157, 158). Curcumin has also been proven to prevent platelet aggregation in atherosclerosis and lower platelet-leukocyte adhesion in cerebral microcirculation (159). Besides, several other natural products, including Grape seed proanthocyanidin extract, Thymol, Kaempferol, and Luteolin, also alleviate psoriasis via reducing the frequency and function of Th17 cells and increasing that of Treg cells (160), and simultaneously inhibit platelet aggregation, inflammatory cell and platelet adhesion, and thrombogenesis (161–164). However, research on the efficacy of natural products in psoriasis and psoriasis-associated CVD is still insufficient, awaiting further studies.

In general, given the important role of platelet activation in psoriasis and CVD, targeting platelets and platelet-derived mediators may be a potent novel strategy for psoriasis treatment in the future.

## 6 Conclusion

Platelets may be excessively activated via endothelial damage, numerous cytokines release and PAF production induced by abnormal immune system in psoriasis, and in turn overactivation of platelets may contribute to initiation and progression of immune responses in lesional skin and blood vessels, ultimately leading to psoriasis and its comorbidities such as atherosclerosis, ischemic heart disease, stroke, MI, and other CVD. Besides, biomarkers of platelet activation which have been well investigated showed a direct relationship between platelet activation and psoriasis. Based on this point, antiplatelet therapies including aspirin and statins might have positive efficacy on psoriasis and psoriasis-associated CVD. Although some encouraging data have been published, deeper studies are urgently warranted to establish the unambiguous role for platelet activation in the pathogenesis of psoriasis and psoriasis-associated CVD. Also, additional large and prospective studies are required to provide compelling evidence regarding good effects of antiplatelet therapies in psoriasis management.

## Author contributions

ZJ reviewed the current literature, wrote the manuscript, and designed the figure. XJ reviewed the literature and drafted the manuscript. AC aided in revising the manuscript. WH devised the structure of the review and wrote the manuscript. All authors contributed to the article and approved the submitted version.
